# The molecular chaperone Hsp90α deficiency causes retinal degeneration by disrupting Golgi organization and vesicle transportation in photoreceptors

**DOI:** 10.1093/jmcb/mjz048

**Published:** 2019-08-13

**Authors:** Yuan Wu, Xiudan Zheng, Yubo Ding, Min Zhou, Zhuang Wei, Tao Liu, Kan Liao

**Affiliations:** 1 Key Laboratory of Systems Biology, CAS Center for Excellence in Molecular Cell Science, Shanghai Institute of Biochemistry and Cell Biology, Chinese Academy of Sciences, University of Chinese Academy of Sciences, Shanghai 200031, China; 2 Department of Ophthalmology, Eye and ENT Hospital of Fudan University, Shanghai 200031, China

**Keywords:** Hsp90α, retinitis pigmentosa, Golgi disintegration, vesicle transportation, MAP1B, acetylated α-tubulin, microtubule, cytoskeleton

## Abstract

Heat shock protein 90 (Hsp90) is an abundant molecular chaperone with two isoforms, Hsp90**α** and Hsp90**β**. Hsp90**β** deficiency causes embryonic lethality, whereas Hsp90**α** deficiency causes few abnormities except male sterility. In this paper, we reported that Hsp90**α** was exclusively expressed in the retina, testis, and brain. Its deficiency caused retinitis pigmentosa (RP), a disease leading to blindness. In Hsp90**α**-deficient mice, the retina was deteriorated and the outer segment of photoreceptor was deformed. Immunofluorescence staining and electron microscopic analysis revealed disintegrated Golgi and aberrant intersegmental vesicle transportation in Hsp90**α**-deficient photoreceptors. Proteomic analysis identified microtubule-associated protein 1B (MAP1B) as an Hsp90**α**-associated protein in photoreceptors. Hsp**α** deficiency increased degradation of MAP1B by inducing its ubiquitination, causing **α**-tubulin deacetylation and microtubule destabilization. Furthermore, the treatment of wild-type mice with 17-DMAG, an Hsp90 inhibitor of geldanamycin derivative, induced the same retinal degeneration as Hsp90**α** deficiency. Taken together, the microtubule destabilization could be the underlying reason for Hsp90**α** deficiency-induced RP.

## Introduction

The 90-kDa heat shock protein (Hsp90) is an abundant and conserved molecular chaperone. It has two isoforms in mammalian cells: the inducible Hsp90α and constitutive Hsp90β (Farrelly and Finkelstein, 1984; Bardwell and Craig, 1987; McDowell et al., 2009). The binding of Hsp90 helps the client proteins to correctly fold or change conformations, leading to their stabilization or activation (Pratt and Toft, 2003). Hsp90 prefers to interact with a specific subset of client proteins and most of them can be classified into two groups: transcription factors and signaling kinases (Picard, 2002). By regulating client proteins, Hsp90 participates in many cellular activities including cell cycle, cell survival, hormone, and other signaling pathways (Picard et al., 1990; Aligue et al., 1994; Dias et al., 2002; Senju et al., 2006). It has been estimated that Hsp90 represents 1%–2% of total cytoplasmic proteins in unstressed cells (Csermely et al., 1998). Its abundance enables Hsp90 to bind to a large number of client proteins in substantial quantity. Until now, most investigations on Hsp90 are focused on its functions in cellular signaling pathways. Its physiological effects on mammals, however, still require further investigation.

Recently, it has been reported that the human clinical trial of 17-DMAG, a synthetic Hsp90 inhibitor of geldanamycin derivative, was halted for its retinal side effects such as night blindness, blurred vision, and flashes (Kummar et al., 2010; Pacey et al., 2011). It was also reported that rats and beagle dogs treated with 17-DMAG or other Hsp90 inhibitors suffered severe retinal photoreceptor degeneration (Zhou et al., 2013; Kanamaru et al., 2014). These findings suggest important physiological functions played by Hsp90 in the retina.

Retinal photoreceptors (rod and cone cells) are polarized sensory neurons with a specialized primary cilium that is called outer segment (OS). A photoreceptor is composed of OS, connecting cilium (CC), inner segment (IS), nucleus, and a synaptic terminus (Sidman, 1957). OS is a photosensitive organelle designed to capture photons and propagate visual signals. It is composed of well-aligned and tightly packed discs in which large amounts of photopigment and phototransduction proteins reside (Molday, 1998). OS develops from CC by receiving proteins and membranes from IS because there are no biosynthetic organelles in OS (Besharse and Horst, 1990). In mammalian retina, the OSs are rapidly turned over, requiring frequent intersegmental transportation to maintain tissue morphology and function (LaVail, 1973). It has been known that defects in intra-photoreceptor transportation lead to photoreceptor degeneration and blindness. The underlying mechanisms, however, are still not fully understood.

A high protein-processing activity is required to sustain the frequent intersegmental protein trafficking and fast OS turnover of photoreceptor. Chaperones are important components to facilitate protein assembly and enhance protein stability in a protein-rich environment. In our current study, we found that Hsp90α deficiency in mice could lead to retinitis pigmentosa (RP), a common inherited retinal disease involving progressive photoreceptor degeneration and the eventual blindness. In retinal photoreceptors, Hsp90α deficiency caused Golgi apparatus disintegration and impaired intersegmental vesicle trafficking. Further analysis suggested that microtubule-associated protein 1B (MAP1B) degradation and microtubule destabilization might be the reason of these phenotypes. Collectively, our results provided a new insight into the causes of RP, and suggested microtubule stabilization might be a potential treatment for RP.

## Results

### The expression of Hsp90α in the retina

**Figure 1:**
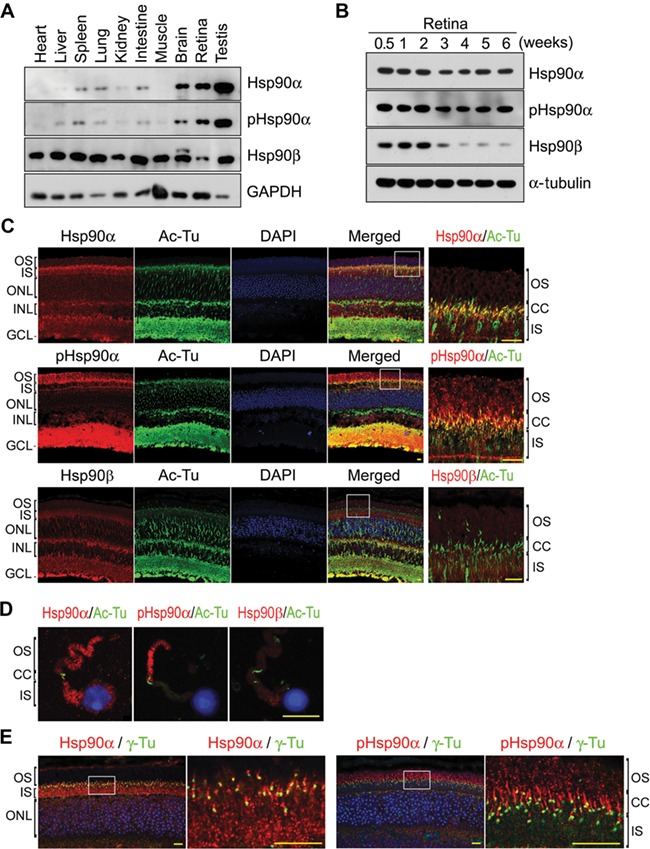
Expression of Hsp90 in the retina. Scale bar, 10 μm. (**A**) The expression of Hsp90α and Hsp90β in mouse tissues. Tissues were isolated from wild-type C57BL/6 mice at 6 weeks of age. An equal amount of protein was loaded for each tissue on SDS–PAGE. (**B**) The expression of Hsp90α and Hsp90β in developing retinas. The retinas were isolated from wild-type C57BL/6 mice at the indicated ages. (**C**) Immunofluorescence staining of mouse retina for Hsp90α and Hsp90β. Retinal sections of wild-type C57BL/6 mice at the age of 6 weeks were stained with antibodies against Hsp90α, Hsp90β, and Thr5/Thr7 phosphorylated Hsp90α (pHsp90α). Acetylated α-tubulin (Ac-Tu) was stained to show CC. The boxed areas are enlarged to show details. (**D**) Immunofluorescence staining for Hsp90α and Hsp90β in rod cells isolated from wild-type C57BL/6 mice at the age of 6 weeks. (**E**) Immunofluorescence staining for Hsp90α and the basal body of CC. Retinal sections were stained for γ-tubulin (γ-Tu) to show basal body. The boxed areas are enlarged to show details.

Hsp90α and Hsp90β are highly homologous with 86% amino acid sequence identity. To ensure the separation of these two isoforms, antibodies against Hsp90α or Hsp90β were tested and two unique phosphorylation sites in N-terminal region (Thr5/Thr7) of Hsp90α were used to differentiate Hsp90α from Hsp90β (Supplementary Figure S1A). Hsp90β was the major Hsp90 isoform in most tissues except the retina, in which Hsp90α was more abundant (Figure 1A). In addition, Hsp90α was predominantly expressed in the testis, brain, and retina, but not in other tissues (Figure 1A).

Mouse retina is not fully developed till 3 weeks after birth (LaVail, 1973). Both Hsp90α and Hsp90β were expressed in the developing retina of neonatal mice. However, the expression of Hsp90β was suppressed after the retina was fully developed, leaving Hsp90α as the major Hsp90 isoform in the developed retina (Figure 1B). Hsp90α was present in both OS and IS of photoreceptors, and it was mostly phosphorylated at Thr5 and Thr7 residues in OS (Figure 1C–E).

### Retinal degeneration and photoreceptor apoptosis in Hsp90α-deficient mice

**Figure 2:**
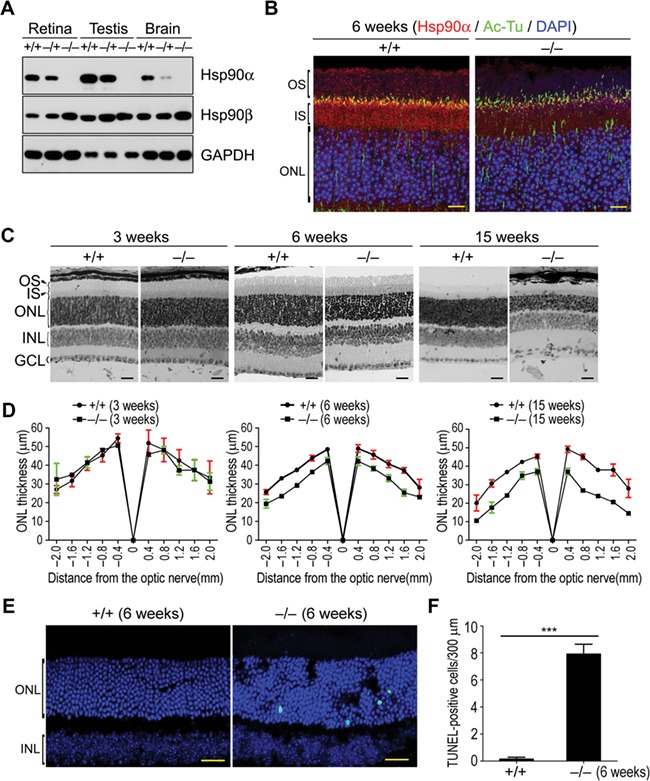
Retinal degeneration and photoreceptor apoptosis in Hsp90α-deficient mice. (**A**) The expression of Hsp90α in wild-type, heterozygous, and homozygous Hsp90α-deficient mice. +/+, −/+, and −/− indicate wild-type mouse, heterozygote, and homozygote, respectively. Mice were 6 weeks old. (**B**) Immunofluorescence staining for Hsp90α on retinal sections of wild-type and homozygous Hsp90α-deficient mice. Scale bar, 10 μm. (**C**) Histological examination of the retina in Hsp90α-deficient mice at different ages (3, 6, and 15 weeks). Scale bar, 20 μm. (**D**) Spider plot of ONL thickness. The retinas of Hsp90α-deficient mice or wild-type littermates were analyzed for ONL thickness. Each point represents mean ± SEM. The data were obtained from three mice at 6 weeks or 15 weeks of age and two mice at 3 weeks of age for each group. (**E**) Photoreceptor apoptosis in Hsp90α-deficient mice. Retinal sections were stained by TUNEL. Scale bar, 25 μm. (**F**) Quantification of TUNEL-positive cells in the retina. Data are mean ± SEM of the results obtained from three mice for each group. ***P < 0.001 (Student’s *t*-test).

To investigate the function of Hsp90α in the retina, Hsp90α-deficient mice were generated by inserting a viral DNA fragment into the ninth exon of *Hsp90α* gene. This insertion caused a frameshift mutation in the coding region of *Hsp90α* (Supplementary Figure S1B). Consequently, Hsp90α translation was terminated and there was no detectable Hsp90α protein in homozygous Hsp90α-deficient mice. Hsp90β level, on the other hand, increased slightly to compensate the loss of Hsp90α (Figure 2A). The absence of Hsp90α in the retina was further verified by immunofluorescence staining for Hsp90α on retinal tissue sections (Figure 2B).

The retina of 3-week Hsp90α-deficient mice showed no signs of degeneration or morphological abnormities in comparison to that of the wild-type littermates. The loss of photoreceptors was detected in the retina of 6-week Hsp90α-deficient mice and the outer nuclear layer (ONL) was much thinner. By the age of 15 weeks, the retina of Hsp90α-deficient mice was completely deteriorated with a massive loss of photoreceptors (Figure 2C and D). These histological observations were verified by TUNEL assay, which revealed photoreceptor apoptosis in the retina of 6-week Hsp90α-deficient mice (Figure 2E and F). This photoreceptor apoptosis was accompanied by the activation of caspase-3 and caspase-9 (Supplementary Figure S2A and B). In newly developed retina of 3-week-old mice, there was no difference in the activation of caspase-3 or caspase-9 between Hsp90α-deficient mice and their wild-type littermates (Supplementary Figure S2A and B). Thus, Hsp90α deficiency did not affect retinal development but induced retinal degeneration by promoting photoreceptor apoptosis.

### Photoreceptor deterioration and loss of retinal functions in Hsp90α-deficient mice

**Figure 3:**
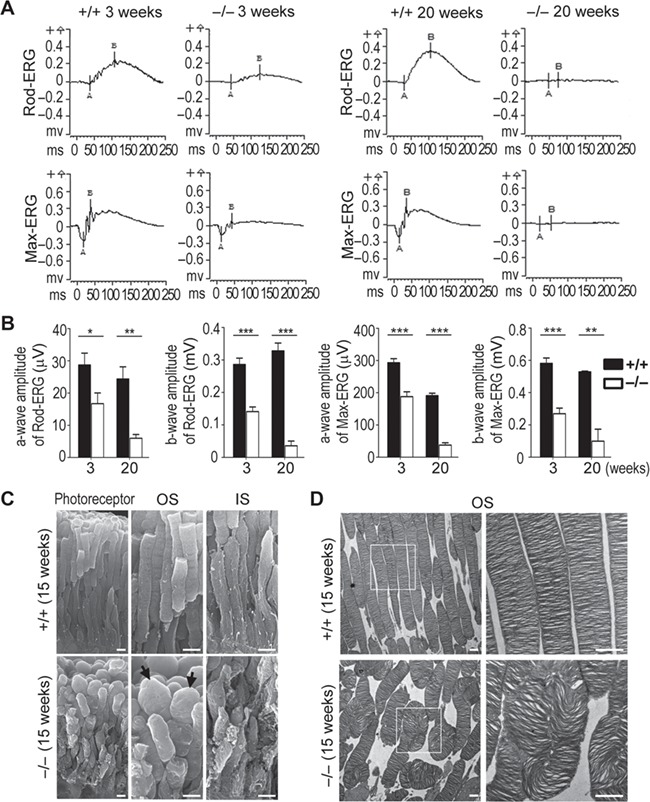
Decreased ERG response and deteriorated photoreceptor in Hsp90α-deficient mice. (**A**) ERG was recorded for Hsp90α-deficient mice or their wild-type littermates at postnatal 3 and 20 weeks. The rod-ERG and max-ERG were recorded in order. (**B**) Statistical analysis of ERG amplitudes. The amplitudes of a-wave and b-wave of rod-ERG and max-ERG were analyzed. Data are mean ± SEM of the results obtained from 10 mice at the age of 3 weeks and 3 mice at 20 weeks for each group. Student’s *t*-test, *P < 0.05, **P < 0.01, ***P < 0.001. (**C**) SEM observation of deformed photoreceptors in 15-week Hsp90α-deficient mice. The black arrows point to the bulged OSs in Hsp90α-deficient mice. Scale bar, 2 μm. (**D**) TEM observation of OS discs in photoreceptors of 15-week Hsp90α-deficient mice. The boxed areas are enlarged to show details. Scale bar, 1 μm.

Electroretinography (ERG) is a non-invasive method to measure the physiological functions of the retina in response to light stimulation. Rod-ERG represents the functions of rod photoreceptors under the stimulation of weak light flashes and max-ERG represents the combined functions of both rod and cone cells in response to the stimulation of standard light flashes (Gundogan et al., 2011; McCulloch et al., 2015). The a-wave of ERG derives from photoreceptors and the b-wave originates from bipolar cells (Hood, 2000). As shown in Figure 3A and B, the ERG amplitudes of 3-week Hsp90α-deficient mice were significantly decreased in comparison to that of the wild-type littermates. The retinal function of Hsp90α-deficient mice was already compromised, even though the retina had yet developed histological abnormities at that age (Figure 2C and D). At 20 weeks, Hsp90α-deficient mice were essentially blind as there was no ERG amplitude in response to light stimulation (Figure 3A and B).

Scanning electron microscopy (SEM) and transmission electron microscopy (TEM) are effective methods to visualize photoreceptor morphology and ultra-structures. In the retina of Hsp90α-deficient mice, the OSs of photoreceptors were no longer in an orderly arranged cylindrical shape but in swollen and deformed shapes, and their membrane discs were twisted and coiled (Figure 3C and D). In addition, the ISs of photoreceptors were also deteriorated with rough membrane surface and deformed shapes (Figure 3C). In other words, the deteriorated and dysfunctional photoreceptors caused retinal dysfunction and degeneration in Hsp90α-deficient mice.

### Disintegrated Golgi apparatus in photoreceptors of Hsp90α-deficient mice

The initial development of the retina in Hsp90α-deficient mice appeared to be normal (Figure 2C and D). Hsp90α deficiency induced photoreceptor degeneration in the developed retina but not in the developing retina, suggesting an impaired turnover of photoreceptor membrane after retinal development. Intersegmental vesicle trafficking is essential for supplying the membranes and proteins to sustain the fast turnover of OS. Any impairment or interference in the intersegmental trafficking would cause the imbalance of OS turnover and lead to photoreceptor deterioration and dysfunction.

**Figure 4:**
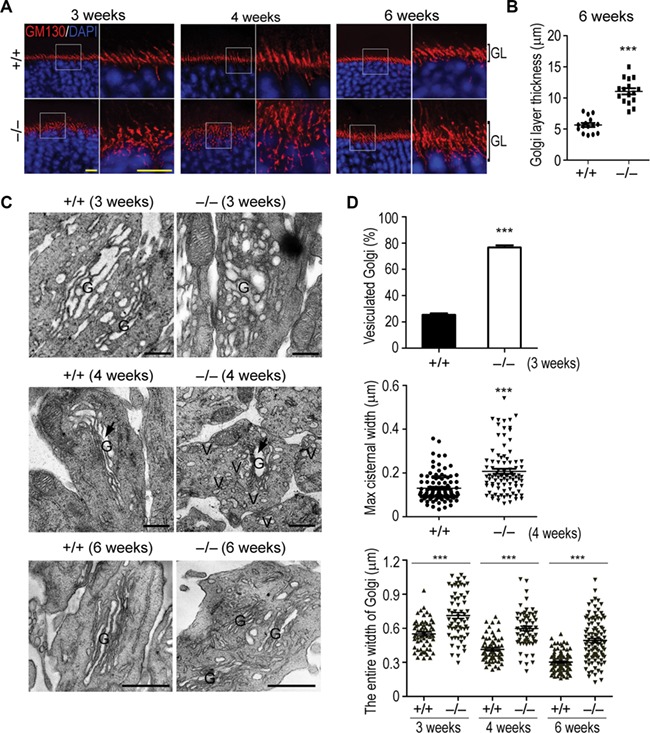
Golgi disintegration in photoreceptors of Hsp90α-deficient mice. (**A**) Golgi apparatus in photoreceptors of Hsp90α-deficient mice. Retinal sections of Hsp90α-deficient mice (*−/−*) or wild-type littermates (*+/+*) were stained for GM130, a cis-Golgi marker. Mouse ages (3, 4, and 6 weeks) are indicated. The boxed areas are enlarged to show details. GL, photoreceptor Golgi layer. Scale bar, 10 μm. (**B**) Quantification of Golgi layer thickness in photoreceptors of 6-week Hsp90α-deficient mice or wild-type littermates. Golgi layer thickness of photoreceptors was measured in three visual fields per mouse and 5 mice were analyzed for each group. Data are mean ± SEM. ***P < 0.001 (Student’s *t*-test). (**C**) TEM observation of Golgi apparatus in photoreceptors of Hsp90α-deficient mice. G, Golgi; V, Golgi-deriving vesicles. The black arrows indicate the maximum cisternal lumen of Golgi in the micrographs. Scale bar, 0.5 μm. (**D**) The statistical analyses of photoreceptor Golgi parameters in wild-type and Hsp90α-deficient mice at the indicated ages. Quantification of vesiculated photoreceptor Golgi in 3-week Hsp90α-deficient or wild-type mice was obtained from 3 mice and at least 108 cells were counted per mouse. Quantification of maximum luminal width of photoreceptor Golgi cisternae in 4-week mice was obtained from 2 mice for each group. Totally 100 cells in wild-type mice and 90 cells in Hsp90α-deficient mice were analyzed. Quantification of entire photoreceptor Golgi width was obtained from 2 mice for each group. Golgi from 63 photoreceptors of 3-week mice, 65 photoreceptors of 4-week mice, and 100 photoreceptors of 6-week mice was analyzed. The entire width of Golgi was measured to show the dispersal degree of photoreceptor Golgi. Data are mean ± SEM. ***P < 0.001 (Student’s *t*-test).

Golgi apparatus is the pivotal organelle in vesicle trafficking and can be detected by the staining for GM130, a marker protein of cis-Golgi. GM130 staining on retinal frozen sections of wild-type mice revealed well-aligned Golgi in IS of photoreceptors (Figure 4A and B). However, the Golgi became misplaced or broken in photoreceptors of Hsp90α-deficient mice and was not confined in IS region (Figure 4A and B). The visualization of Golgi ultra-structure by TEM analysis showed compromised Golgi stacks in Hsp90α-deficient photoreceptors. They were disintegrated and distributed in a larger cytoplasmic area (Figure 4C and D). In fact, the Golgi disintegration and expansion in photoreceptors had already occurred in Hsp90α-deficient mice at 3 weeks of age, well before the onset of retinal degeneration (Figure 4C). This trend of Golgi disintegration continued in older ages and was validated by measuring the parameters of Golgi (Figure 4C and D).

### Aberrant rhodopsin transport in photoreceptors of Hsp90α-deficient mice

**Figure 5:**
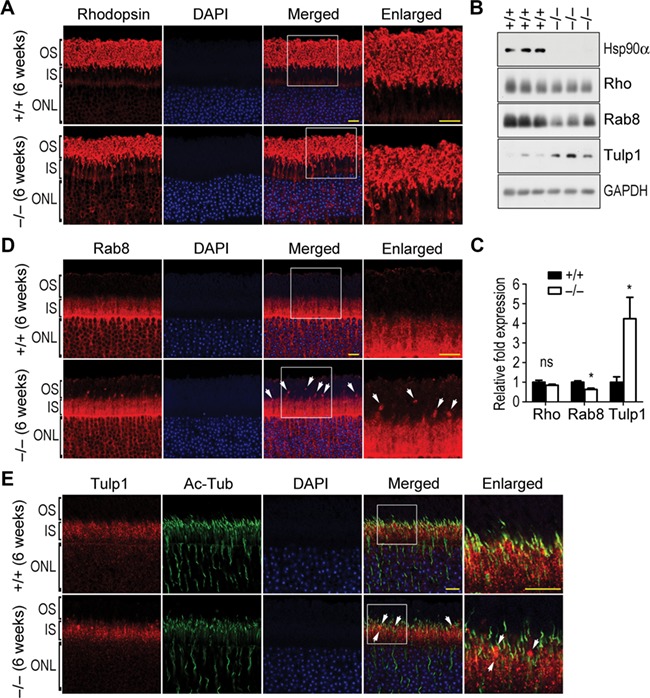
Abnormal rhodopsin transport in Hsp90α-deficient mice. Scale bar, 10 μm. The boxed areas are enlarged to show details. (**A**) The retention of rhodopsin in IS and ONL of photoreceptors in 6-week Hsp90α-deficient mice. (**B**) The expression of rhodopsin (Rho), Rab8, and Tulp1 in the retinas of Hsp90α-deficient mice or wild-type littermates. GAPDH was the protein loading control. (**C**) Quantification of protein expression. Rhodopsin, Rab8, and Tulp1 on western blot were subjected to densitometry analyses and normalized by the loading control GAPDH. Fold expression of the proteins was calculated for Hsp90α deficiency relative to the wild-type. Data are expressed as mean ± SEM of the results obtained from three mice. *P < 0.05 (Student’s *t*-test). (**D**) Aggregated Rab8 in photoreceptors of Hsp90α-deficient mice. Arrows indicate the Rab8 clusters. (**E**) Aggregated Tulp1 along CC of photoreceptors in Hsp90α-deficient mice. Arrows indicate the Tulp1 clusters.

Rhodopsin is the major protein in OS of photoreceptor and accounts for ~80% of the proteins in OS membrane (Heitzmann, 1972). It is synthesized in IS and exclusively delivered to OS. In wild-type mice, no rhodopsin was retained in IS of photoreceptor. In Hsp90α-deficient mice, however, a substantial amount of rhodopsin was aberrantly retained in IS of photoreceptor though the overall rhodopsin synthesis was not affected by the absence of Hsp90α (Figure 5A–C). It was the transportation of rhodopsin from IS to OS that was impaired by the lack of Hsp90α. The retention of rhodopsin in IS was resulted from Golgi disintegration and both were detected in Hsp90α-deficient mice of 3 weeks old, prior to the onset of photoreceptor deterioration (Figure 4C; Supplementary Figure S3).

Rab8, a small GTPase that is involved in rhodopsin transport and vesicular trafficking (Deretic et al., 1995; OL et al., 2001), aggregated abnormally in the region between the IS and OS of Hsp90α-deficient photoreceptors (Figure 5D). In addition, Tulp1, which is reported to participate in rhodopsin transportation as well as polarized vesicle trafficking (Hagstrom et al., 1999; Grossman et al., 2011), had been identified to interact with Hsp90α in photoreceptor by mass spectrometry analysis (Supplementary Figure S4D). It also clustered aberrantly along the CC region of photoreceptor in Hsp90α-deficient mice (Figure 5E).

Golgi apparatus is the key organelle in post-translational protein process and transportation. The combination of disintegrated Golgi and aberrant rhodopsin transportation in Hsp90α-deficient photoreceptor suggested a probable cause for the retinal degeneration. In other words, the disintegrated Golgi affected the intersegmental protein transportation that is essential for turnover of photoreceptor membrane. Subsequently, photoreceptors gradually lost their structural integrity, leading to complete retinal dysfunction of Hsp90α-deficient mice.

### MAP1B reduction and microtubule destabilization in Hsp90α-deficient photoreceptors

**Figure 6:**
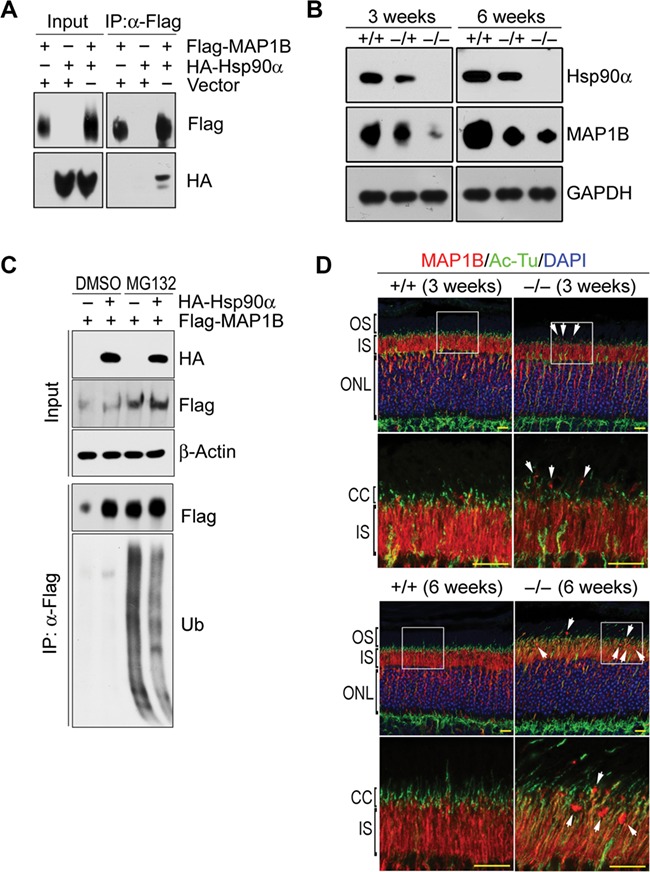
MAP1B reduction in the retina of Hsp90α-deficient mice. (**A**) Interaction between Hsp90α and MAP1B. HA-tagged Hsp90α and Flag-tagged MAP1B were co-expressed in HEK293T cells and MAP1B was immunoprecipitated by anti-Flag affinity gel. (**B**) MAP1B reduction in the retina of Hsp90α-deficient mice. The retinas of wild-type (*+/+*), heterozygous (*−/+*), and homozygous (*−/−*) Hsp90α-deficient mice at the age of 3  or 6 weeks were isolated for western blot. (**C**) Decreased MAP1B ubiquitination by Hsp90α co-expression. Flag-tagged MAP1B was expressed in HEK293T cells with or without the co-expression of HA-tagged Hsp90α. MAP1B ubiquitination was analyzed after cells were treated with proteasome inhibitor MG132. Flag-tagged MAP1B was immunoprecipitated by anti-Flag affinity gel (Flag) and blotted by anti-ubiquitin antibody (Ub). (**D**) Immunofluorescence staining for MAP1B on retinal sections of wild-type (*+/+*) or Hsp90α-deficient mice (*−/−*) at the age of 3  or 6 weeks, respectively. The boxed areas are enlarged to show details. Arrows indicate MAP1B clusters. Scale bar, 10 μm.

Cellular microtubules are scaffolds to support intracellular membrane systems and maintain the structural integrity of Golgi apparatus (Rogalski and Singer, 1984; Thyberg and Moskalewski, 1999). MAP1B is a microtubule-associated protein with neuronal distribution and functions to stabilize microtubule by promoting α-tubulin acetylation (Takemura et al., 1992; Ryan et al., 2012). As shown in Supplementary Figure S4, MAP1B was among the major Hsp90α-associated proteins in the retina. And their interaction was confirmed by the association of exogenously expressed MAP1B and Hsp90α (Figure 6A). In addition, the presence of HA-tagged Hsp90α increased and stabilized the co-expressed Flag-tagged MAP1B (Figure 6A). It greatly reduced MAP1B ubiquitination (Figure 6C). Thus, Hsp90α could stabilize MAP1B by preventing its ubiquitination and degradation. In Hsp90α-deficient mice, the absence of Hsp90α in the retina caused dramatic reduction of MAP1B protein level (Figure 6B). The stabilization of MAP1B by Hsp90α binding implicated that Hsp90α deficiency affected microtubule system in the retina.

**Figure 7:**
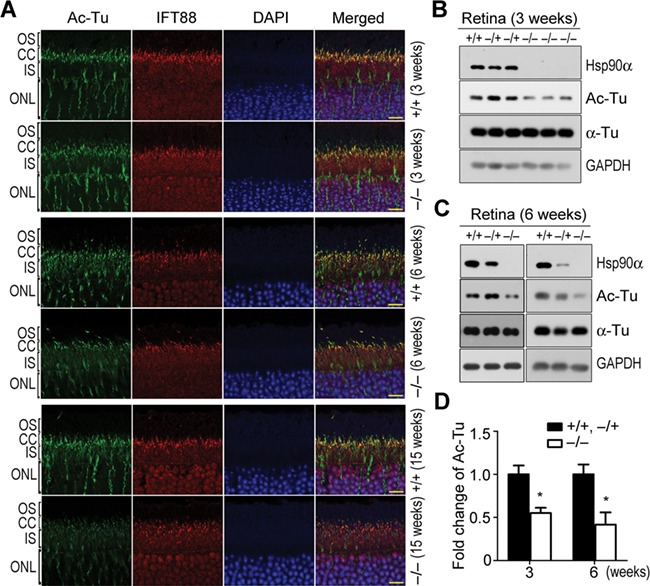
α-tubulin deacetylation and microtubule destabilization in photoreceptors of Hsp90α-deficient mice. (**A**) α-tubulin deacetylation in photoreceptors of Hsp90α-deficient mice. IFT88 was stained on retinal sections to show the CC. Scale bar, 10 μm. (**B** and **C**) Analysis of α-tubulin deacetylation by western blot. The retinas of wild-type (*+/+*), heterozygous (*−/+*), and homozygous (*−/−*) Hsp90α-deficient mice at the age of 3 or 6 weeks were isolated for western blot. (**D**) Quantification of acetylated α-tubulin. Acetylated α-tubulin on western blot was subjected to densitometry analysis and normalized by total α-tubulin. Fold expression of acetylated α-tubulin was calculated for Hsp90α deficiency relative to wild-type and heterozygote. Data are mean ± SEM of the results obtained from at least two mice for each group. *P < 0.05 (Student’s *t*-test).

In Hsp90α-deficient mice, the CC of photoreceptor was misplaced and less organized (Figure 6D). There were also MAP1B aggregates around the CC region (Figure 6D). IFT88 is one of the intraflagellar transport proteins associated with cilium for transporting protein. In the retina, both the CC and its associated IFT88 were affected by Hsp90α deficiency. There was less tubulin acetylation or IFT88 association (Figure 7A). The absence of Hsp90α also caused the overall reduction of α-tubulin acetylation in the retina (Figure 7B–D). Microtubule acetylation stabilizes the cytoskeleton and enables it to be more resistant to depolymerizing conditions or mechanical breakage (Piperno et al., 1987; Xu et al., 2017). The microtubules around Golgi are mostly acetylated forms, which are essential for Golgi organization and vesicle trafficking (Thyberg and Moskalewski, 1993; Ryan et al., 2012). The reduction of MAP1B and microtubule acetylation in Hsp90α-deficient photoreceptor was most likely the major factor to cause Golgi disintegration.

### Retinal degeneration induced by 17-DMAG, an inhibitor of Hsp90

**Figure 8:**
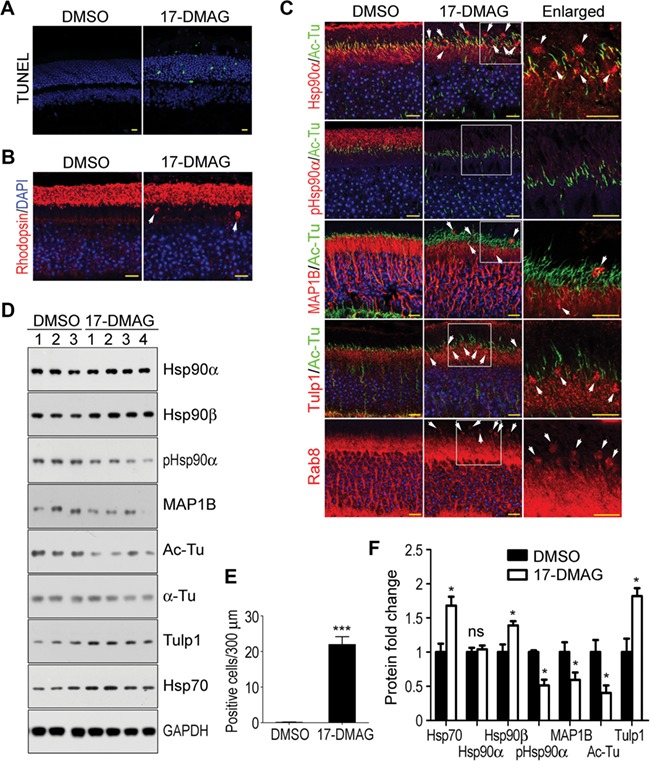
Retinal degeneration of wild-type mice induced by 17-DMAG treatment. Scale bar, 10 μm. (**A**) Photoreceptor apoptosis in the retina of 17-DMAG-treated mice. The cell apoptosis was detected by TUNEL staining. (**B**) Rhodopsin retention in IS of 17-DMAG-treated mice. The white arrows indicate the mislocated rhodopsin in IS. (**C**) Abnormal localization and aggregation of Hsp90α, MAP1B, Rab8, and Tulp1 in photoreceptors of 17-DMAG-treated mice. The boxed areas are enlarged to show details and the white arrows indicate the protein clusters. (**D**) MAP1B reduction and α-tubulin deacetylation in the retinas of 17-DMAG-treated mice. The retinas of DMSO- or 17-DMAG-treated mice were isolated for western blot. The numbers indicated individual mouse treated with DMSO or 17-DMAG. (**E**) Quantification of TUNEL-positive cells in the retina. Data are mean ± SEM of the results obtained from three mice for each group. ***P < 0.001 (Student’s *t*-test). (**F**) Quantification of protein expression in the retina of 17-DMAG-treated mice. Protein bands on western blot were subjected to densitometry analyses and normalized by GAPDH. Fold expression of the proteins was calculated for 17-DMAG relative to DMSO. Data are mean ± SEM of the results obtained from two independent experiments. *P < 0.05 (Student’s *t*-test).

As Hsp90α deficiency induced retinal degeneration, the inhibition of Hsp90α should induce a similar effect on the retina. 17-DMAG, an Hsp90 inhibitor that is capable of passing blood-retina barrier (Zhou et al., 2013), was used to inject mice. In the retina, the inhibition of Hsp90 by 17-DMAG treatment was verified by the upregulation of Hsp70, a biomarker for Hsp90 inhibition (Whitesell et al., 2003) (Figure 8D and F). The treatment of wild-type mice with 17-DMAG for more than a week induced photoreceptor apoptosis similar to that in Hsp90α-deficient mice (Figure 8A and E). The molecular alterations of photoreceptor caused by Hsp90α absence, such as the rhodopsin retention in IS and the clustering of Rab8, Tulp1, and MAP1B, were all induced by 17-DMAG treatment in wild-type mice (Figures 5 and 8B, C). Furthermore, 17-DMAG also induced MAP1B reduction and α-tubulin deacetylation (Figure 8D and F). Overall, the pathological effects of 17-DMAG treatment on the retina were the same as the phenotypes of Hsp90α deficiency.

Hsp90 has been a promising anti-tumor target and several Hsp90 inhibitors have been used in clinical trials (Goldman et al., 2010; Demetri et al., 2011; Dickson et al., 2012). It was reported that 17-DMAG could cause retinal side effects such as night blindness, blurred visions, and flashes in human anti-tumor clinical trials (Kummar et al., 2010; Pacey et al., 2011). These side effects on the retina by 17-DMAG further supported that Hsp90α is important for the well beings of photoreceptor and its physiological functions.

## Discussion

RP is a common blindness-causing disease involving progressive degeneration of photoreceptors or retinal pigment epithelia. It has a worldwide prevalence of 1 in 4000 people, affecting ~1.5 million people worldwide (Ayuso and Millan, 2010). The clinical symptoms of RP are initially night blindness, then the gradual loss of peripheral vision and central vision, and finally blindness (Daiger et al., 2013). Currently, there is no effective drug or method to treat this disease. Mutations in >50 genes have been identified to cause RP (Ferrari et al., 2011). Of these genes, there are proteins in visual transduction cascade such as rhodopsin and PDE, OS structural proteins such as CNGB1, peripherin-2, and ROM1, and vesicular transport proteins such as IFT88, Tulp1, FAM161A, RP1, RP2, and kinesin-2 (Sanyal and Jansen, 1981; Kajiwara et al., 1994; Danciger et al., 1995; Hagstrom et al., 1999; Hardcastle et al., 1999; Lem et al., 1999; Pazour et al., 2002; Jimeno et al., 2006; Zhang et al., 2009; Bandah-Rozenfeld et al., 2010; Grossman et al., 2011). Among the vesicular transport proteins, mutations of RP2 or kinesin-2 are reported to cause Golgi disintegration, similar to Hsp90α deficiency (Stauber et al., 2006; Evans et al., 2010). In addition, FAM161A and RP1, the RP-causing proteins, are microtubule-associated proteins that promote microtubule acetylation and stabilization (Liu et al., 2004; Zach et al., 2012). In our current study of Hsp90α deficiency, microtubule destabilization and Golgi disintegration were identified to cause RP (Figures 4 and 7). Thus, microtubule stabilization could be a novel method for RP treatment.

Night blindness and blurred vision, the early symptoms of RP, are reported as side effects in human anti-tumor clinical trials of 17-DMAG, a synthetic Hsp90 inhibitor of geldanamycin derivative (Kummar et al., 2010; Pacey et al., 2011). Animal experiments reveal 17-DMAG and other Hsp90 inhibitors can induce severe damage to photoreceptors (Zhou et al., 2013; Kanamaru et al., 2014). The effects of 17-DMAG treatment on the retina of wild-type mice and the phenotypes of Hsp90α-deficient mice supported each other (Figures 2, 5, and 8). In both cases, there are photoreceptor apoptosis, rhodopsin retention, and MAP1B clustering. Since Hsp90 has been a promising anti-tumor target and several Hsp90 inhibitors have been used in clinical trials (Goldman et al., 2010; Demetri et al., 2011; Dickson et al., 2012), the function of Hsp90α in the retina raises concern for developing drugs indiscriminatingly targeting both Hsp90 isoforms.

Unlike the constitutive Hsp90β, Hsp90α is the inducible isoform of Hsp90 with restricted distribution in the retina, testis, and brain (Figure 1A). Due to its limited tissue expression profile, its function is often overlooked during the study of Hsp90. The study of gene-deficient mice reveals the functional difference between Hsp90α and Hsp90β. Hsp90β-deficient mice are embryonic lethal (Voss et al., 2000), while Hsp90α-deficient mice look healthy and normal in appearance except for male sterility and RP in our current investigation (Grad et al., 2010; Kajiwara et al., 2012). The male sterility and RP of Hsp90α-deficient mice are well correlated to the expression of Hsp90α in the testis and retina (Figure 1A). The testis of Hsp90α-deficient mice is structurally developed but not functionally mature (Grad et al., 2010; Kajiwara et al., 2012). Similarly, the retina and photoreceptors were developed in Hsp90α-deficient mice but functionally compromised (Figures 2 and 3). It is possible that Hsp90β may play the major role in the development of these two organs, while Hsp90α is important in physiological functions. In addition, the expression of Hsp90α in the brain implicates its possible functions in brain physiology (Figure 1A). 

Golgi elements in the interphase cell are grouped around the stable microtubules such as acetylated or detyrosinated microtubules. The stable microtubules are indispensable for Golgi organization (Thyberg and Moskalewski, 1993). Treatment of cultured cells with nocodazole, a microtubule-depolymerizing reagent, can lead to Golgi fragmentation with dispersed mini-stacks and dilated cisternae, very similar to those alterations of Golgi apparatus in Hsp90α-deficient photoreceptors (Figure 4) (Rogalski and Singer, 1984; Thyberg and Moskalewski, 1999). Microtubule-associated proteins such as neuronal MAP1B, MAP2, tau, and ubiquitously distributed MAP4 often stabilize microtubules by maintaining tubulin acetylation (Takemura et al., 1992; Hirokawa, 1994; Nguyen et al., 1997; Bouquet et al., 2004; Ryan et al., 2012). MAP1B reduction causes Golgi fragmentation and ectopic vesicle transport due to decreased microtubule acetylation, and its heterozygous knockout mice exhibit retinal degeneration with photoreceptor loss (Edelmann et al., 1996; Ryan et al., 2012). These two observations are identical to the retinal phenotypes of Hsp90α-deficient mice (Figures 2–4). Furthermore, MAP1B was downregulated in Hsp90α-deficient photoreceptors (Figure 6). Thus, it is most likely that MAP1B reduction is the fundamental cause for Golgi disintegration, aberrant intersegmental transportation, and photoreceptor degeneration in Hsp90α-deficient mice.

## Materials and methods

### Antibodies and reagents

Anti-Hsp90α (ab74248), anti-Hsp90β (ab2927), and anti-Rhodopsin (ab5417) antibodies were from Abcam. Anti-pHsp90α (T5/7) (#3488) antibody was from Cell Signaling Technology. Anti-acetylated α-tubulin (sc-23950) and anti-Ub (sc8017) antibodies were from Santa Cruz. Anti-Rab8 (610844) and anti-GM130 (610822) antibodies were from BD Pharmingen. Anti-Hsp70 (ADI-SPA-810-F) antibody was from Enzo. Anti-γ-tubulin (T6557), anti-α-tubulin (T5168), anti-GAPDH (G8795), anti-Tulp1 (SAB2700274), anti-MAP1B (HPA022275) antibodies, horse radish peroxidase-conjugated secondary antibody, and DAPI were from Sigma. Alexa-Fluor 488- or 546-conjugated secondary antibodies and anti-fade mounting medium were from Molecular Probes/Invitrogen. TUNEL FITC Apoptosis Detection Kit was from Vazyme. 17-DMAG was from Selleck. Protease and phosphatase inhibitors, Protein A/G magnetic beads, and Anti-Flag affinity gel were from Biomake.

### Animal experiments

The sperms of heterozygous Hsp90α-deficient mice in FVB/NJ background were obtained from MMRRC (036273-JAX). After *in vitro* fertilization, the FVB/NJ Hsp90α-deficient heterozygotes were obtained. The mice were crossbred with wild-type C57BL/6 mice to introduce the knockout *Hsp90α* into C57BL/6 mice and eliminate the *Pdeb^rd1^* mutation carried by FVB/NJ strain. *Pdeb^rd1^* mutation causes rapid photoreceptor death before postnatal 3 weeks, affecting our investigation on Hsp90α and the retina (Yang et al., 2015). C57BL/6 Hsp90α-deficient heterozygotes were genotyped to ensure homozygous wild-type *Pdeb* according to the primer sequences reported (Gimenez and Montoliu, 2001). Homozygous Hsp90α-deficient C57BL/6 mice were obtained for experimentation by crossing the heterozygotes. The genotyping results were shown in Supplementary Figure S1C.

For examination of Hsp90 inhibitor-induced photoreceptor degeneration, wild-type male C57BL/6 mice at postnatal 6 weeks were intravenously injected with 17-DMAG at a dosage of 40 mg/kg or the vehicle DMSO every other day for four times. The animals were sacrificed for analyzing 24 h after the last injection.

All the mice were kept in the animal facility of Shanghai Institute of Biochemistry and Cell Biology, and all the animal experiments were performed in strict accordance with the guidelines of the Institutional Animal Care and Use Committee.

### Light microscopic observation and ONL thickness measurement

Mouse eyes were incised in the cornea and fixed in Carnoy’s solution overnight. After dehydration in N-butanol for 3 days, the cornea and lens were cut off. The remaining eyecup was paraffin infiltrated, embedded, and sectioned in 5-μm thickness. The paraffin sections were deparaffinized, rehydrated, stained with HE, and photographed under Olympus BX51 microscope. ONL thickness measurements were made on digital images of retinal sections every 400 μm from the optic nerve outwards for both the inferior and superior hemisphere by the ruler tool in Photoshop software.

### TUNEL Assays

Mouse eyecup was fixed in 4% paraformaldehyde solution overnight, dehydrated in 10% and 20% sucrose solution for 2 h, respectively, and 30% sucrose solution overnight at 4°C, embedded in Jung Tissue freezing medium (Leica) and then sectioned by freezing microtome. TUNEL FITC Apoptosis Detection Kit (Vazyme) was used to detect cell apoptosis according to the manufacture’s instruction.

### ERG

The ERG tracing and the parameter setting were performed following the reported methods (Gu et al., 2017). The rod-ERG and max-ERG were recorded in order.

### Electron microscopy

The retina from the eyeball was cut into several pieces and fixed in 2.5% glutaraldehyde overnight at 4°C. After washing with phosphate-buffered saline three times, it was postfixed in 1% osmium tetroxide for 2 h at room temperature. The fixed retina was then subjected to dehydration with a series of alcohol and acetone and finally embedded in Epon 812 ethoxyline. The thin section of embedded retina was stained with 2% uranyl acetate and lead citrate and observed under FEI Tecnai G2 Spirit transmission electron microscope.

For SEM, the sample fixation and alcohol dehydration procedures were the same as that for TEM. After dehydration with 100% alcohol, the sample was critical point dried and spurted for observation.

### Immunofluorescence

The retinal paraffin sections were deparaffinized, rehydrated, and boiled in 1 mM EDTA solution (PH = 8.0) for 10 min at 121°C to unmask antigen. Then the retinal sections were incubated with 0.1% Tritonx-100 and 3% BSA dissolved in 0.05% Tween-20 Tris-buffered saline (PH = 7.5) for 1 h at room temperature followed by primary antibody, fluorescein-conjugated secondary antibody, and DAPI solution. The sections were mounted with anti-fade mounting medium and observed with Leica TCS SP8 confocal microscope.

For the immunofluorescence staining of retinal frozen sections, the sections were immersed into boiled 1-mM EDTA solution (pH = 8.0) for 30 min and the rest procedures were the same as those for retinal paraffin sections.

### Quantifications of Golgi alteration in photoreceptor of Hsp90α-deficient mice

For the measurement of Golgi layer thickness, retinal sections of 6-week Hsp90α-deficient mice and their wild-type littermates were stained with anti-GM130 antibody (610822, BD Pharmingen) at a dilution of 1:250. The stained sections were observed under confocal microscope and photographed. The Golgi layer thickness of photoreceptors was measured on microscopic pictures by Leica Application Suite software.

For quantification of vesiculated Golgi of photoreceptors on TEM images, the Golgi apparatus without cisternae was counted as vesiculated and the counting was performed by the count tool of Photoshop software. The number of normal or vesiculated Golgi in each image was typed into an excel sheet and finally the ratio was calculated. The maximum cisternal width and the entire width of Golgi were measured on TEM images with the ruler tool of Photoshop software. The images were counted or measured without previously knowing their labeling of wild-type or knockout mice.

The Golgi of photoreceptors in Hsp90α-deficient mice at different ages was all less compacted, exhibiting certain degrees of disintegration and expansion. The rate of vesiculated Golgi, maximum cisternal width, and entire width of Golgi were parameters to determine the degrees of Golgi compactness and best to represent the detailed Golgi morphology at the corresponding ages.

### Plasmid construction, protein interactions, and ubiquitination experiments

Mouse MAP1B cDNA cloned from the retina of wild-type mouse and mouse Hsp90α cDNA cloned from 3T3-L1 cells were constructed into pCMV-Tag2B or pCDNA3.0 vectors and confirmed by DNA sequencing.

HA-tagged Hsp90α and Flag-tagged MAP1B plasmids were transfected into HEK293T cells by Lipofectamine™ 2000 Transfection Reagent (Thermo Fisher Scientific). Forty-eight hours after transfection, cells were lysed with 0.5% Triton X-100 immunoprecipitation buffer. Flag-tagged MAP1B was precipitated by anti-Flag affinity gel. The samples were analyzed by western blot.

For ubiquitination experiment, 42 h after transfection with HA-tagged Hsp90α and Flag-tagged MAP1B plasmids, HEK293T cells were incubated with 20 μM MG132 (Selleck) or DMSO for 6 h. The cells were harvested and anti-Flag affinity gel was used to precipitate Flag-tagged MAP1B. The ubiquitination was detected by anti-ubiquitin antibody on western blot.

## Acknowledgements

We appreciate Fengling Qin and Fangjie Qi (Shanghai Institute of Biochemistry and Cell Biology) for providing technical support on electron microscopy and the core facility for cell biology for cell imaging.

## Funding

This work was supported by the grant from the National Natural Science Foundation of China (31571387).

**Conflict of interest:** none declared.

## Supplementary Material

JMCB-2018-0422-Supplementary_Material_mjz048
