# Supplementary material for: The molecular chaperone Hsp90α deficiency causes retinal degeneration by disrupting Golgi organization and vesicle transportation in photoreceptors
Source: J Mol Cell Biol. 2019 Aug 13;12(3):216–29. doi: 10.1093/jmcb/mjz048 (PMC7181719; doi:10.1093/jmcb/mjz048)
Supplement: JMCB-2018-0422-Supplementary_Material_mjz048 [file jmcb-2018-0422-supplementary_material_mjz048.pdf]

Supplementary Material

The molecular chaperone Hsp90α deficiency causes retinal degeneration by disrupting Golgi organization and vesicle transportation in photoreceptors

Yuan Wu[1], Xiudan Zheng[1], Yubo Ding[1], Min Zhou[2], Zhuang Wei[1], Tao Liu[1], and Kan Liao[1*]

[1]Key Laboratory of Systems Biology, CAS Center for Excellence in Molecular Cell Science, Shanghai Institute of Biochemistry and Cell Biology, Chinese Academy of Sciences, University of Chinese Academy of Sciences, Shanghai 200031, China

[2]Department of Ophthalmology, Eye and ENT Hospital of Fudan University, Shanghai 200031, China

*Correspondence should be addressed to Kan Liao (kliao@sibs.ac.cn)

Contents

Supplemental Figures 3

Supplemental Figure S-1. The verification of antibodies against Hsp90 and genotyping of Hsp90α-deficient mice 3

Supplemental Figure S-2. The activation of caspase-3 and caspase-9 in retina of Hsp90α-deficient mice 4

Supplemental Figure S-3. Retention of rhodopsin in IS of photoreceptors in 3-week Hsp90α-deficient mice 5

Supplemental Figure S-4. The proteomic analysis of Hsp90α-associated proteins in retinal photoreceptors 6

Reference 7

Supplemental Figures:

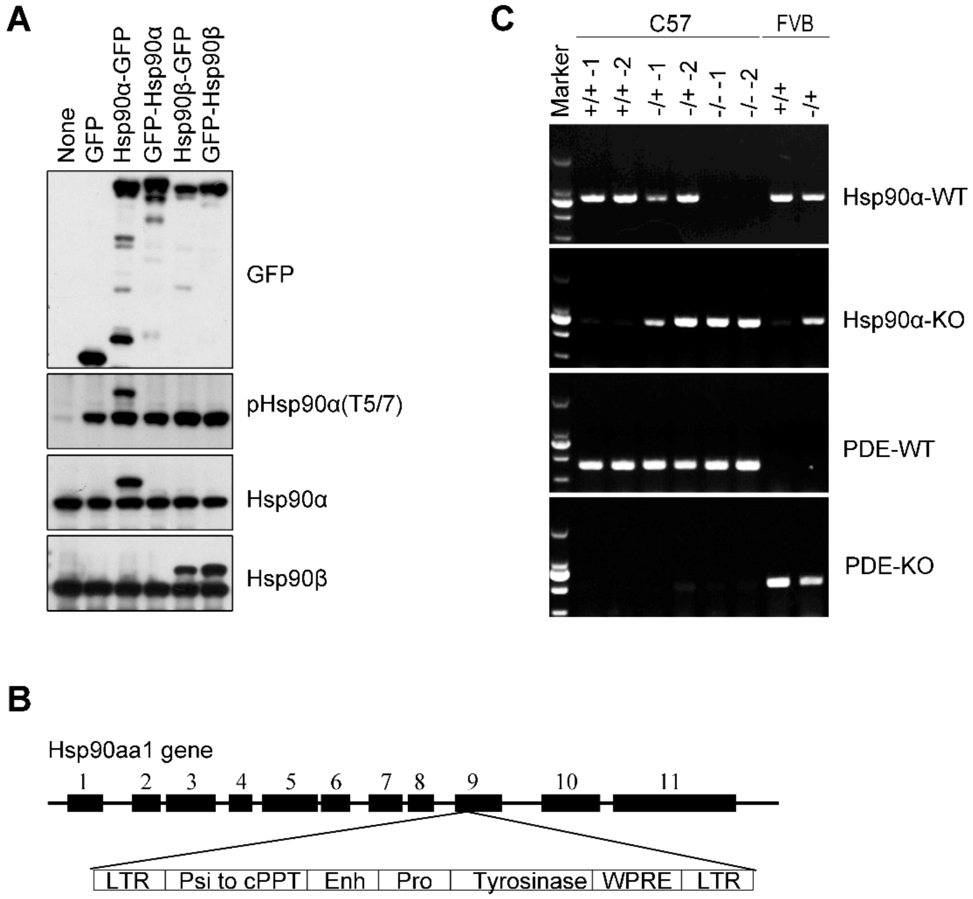

Supplemental Figure S-1. The verification of antibodies against Hsp90 and genotyping of Hsp90α-deficient mice. A. Hsp90α or Hsp90β with GFP tagging at either the N terminus or C terminus was exogenously expressed in HEK293T cells. The specificity of anti-Hsp90α and anti-Hsp90β antibodies was verified by their detection of the GFP-tagged Hsp90α and Hsp90β. As the anti-Hsp90α and anti-Thr5/7 phosphorylated Hsp90α antibodies were produced according to the immunogen peptide corresponding to the 2-12 aa of Hsp90α, their detection of GFP-Hsp90α was blocked by the N-terminally tagged GFP. **B**. Schematic illustration of *Hsp90α* gene disruption by insertion of a viral DNA cassette in the 9th exon of *Hsp90α*. The viral DNA cassette includes these elements: *LTR*, long terminal repeat; *Psi*, packaging sequence; *cPPT*, central polypurine tract; *Enh*, tyrosinase enhancer; *Pro*, tyrosinase promoter; *WPRE*, woodchuck hepatitis virus post-transcriptional regulatory element. **C**. The genotyping of *Hsp90α* and *PDE*. The Hsp90α-deficient mice were originally generated in FVB/NJ strain, which carries the blindness-causing $Pdeb^{rd1}$ mutation. The FVB/NJ Hsp90α-deficient mice were crossbred into C57BL/6 background by crossing with C57BL/6 mice. The final C57BL/6 Hsp90α-deficient mice carried wild-type *PDE* and disrupted *Hsp90α*.

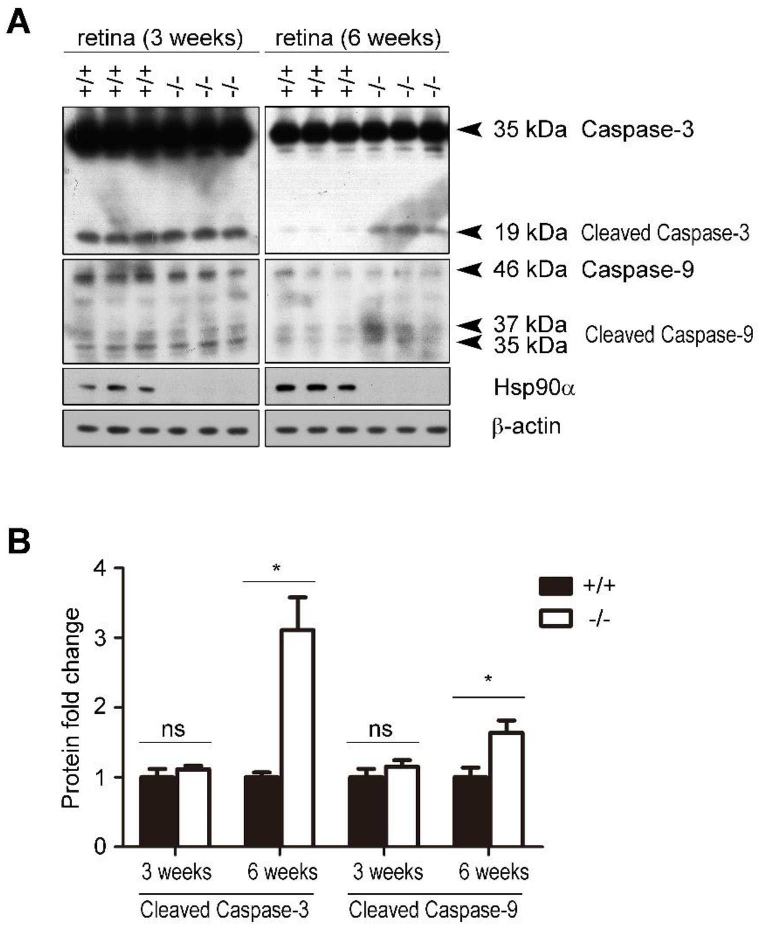

Supplemental Figure S-2. The activation of caspase-3 and caspase-9 in retina of Hsp90α-deficient mice. A. The expression of activated caspase-3 and caspase-9 in retinas of 3-week and 6-week Hsp90α-deficient mice. Initiator caspase (caspase-9) cleaves and activates downstream effector caspase (caspase-3), which in turn induces cell apoptosis by cleaving target proteins. These two caspases were not activated in retina of 3-week Hsp90α-deficient mice, indicating the photoreceptor apoptosis in Hsp90α-deficient mice occurred after retinal development. **B.** Quantification of activated caspase-3 and caspase-9 in retina of Hsp90α-deficient mice. The activated caspase-3 and caspase-9 on Western blot were subjected to densitometry analyses and normalized by the loading control β-actin. Fold expression of the proteins was calculated for Hsp90α deficiency relative to wild type. Data are mean ± SEM of the results obtained from 3 mice for each group. * $P<0.05$ (Student's t-test).

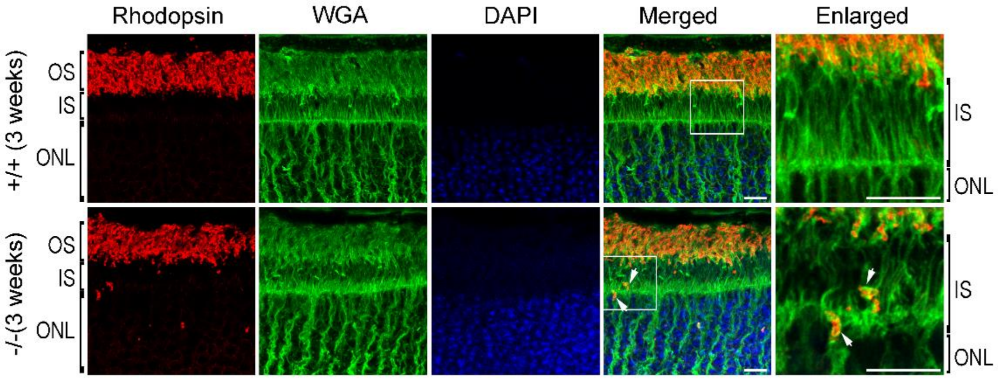

Supplemental Figure S-3. Retention of rhodopsin in IS of photoreceptors in 3-week Hsp90α-deficient mice. The retinal paraffin sections of 3-week mice were stained with anti-rhodopsin antibody and wheat germ agglutinin (WGA), a lectin bound to vesicle membrane. The arrows indicate the rhodopsin clusters packed in vesicles in IS. The boxed areas are enlarged to show details. The bar is 10 μm.

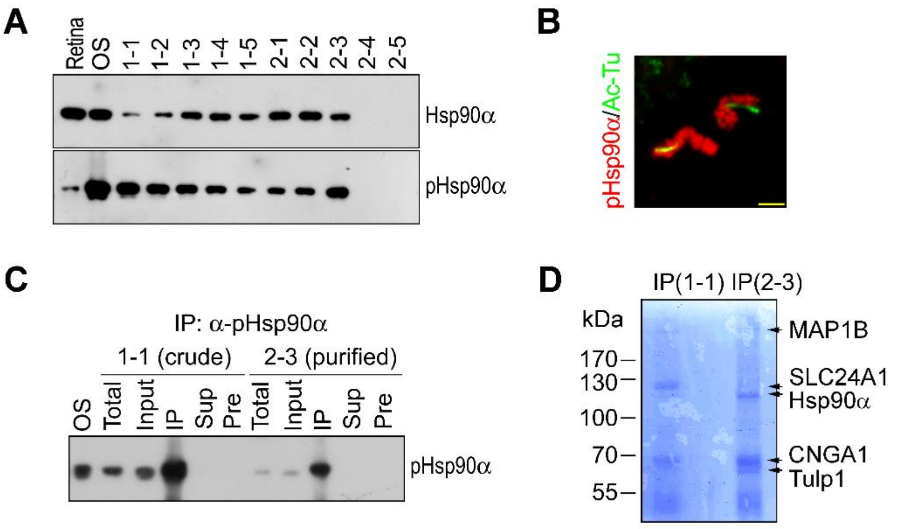

Supplemental Figure S-4. The proteomic analysis of Hsp90α-associated proteins in retinal photoreceptors. **A**. Isolation of photoreceptor OS. The photoreceptor sensing cilium complexes (PSCC), containing OS and CC, were isolated from 6-week wild-type C57BL/6 mice following the reported methods (Liu et al., 2007). *Retina*, total retinal lysate of wild-type C57BL/6 mice; *OS*, crude OSs directly shaken off from retina. The crude OSs were adjusted to high sucrose concentration and centrifuged. *1-1, 1-2……1-5* indicate the floated fractions from top to bottom. 1-1 was the fraction at the top and abundant of crude OSs. Fraction 1-1 was diluted, applied to a dense sucrose cushion and then centrifuged. *2-1, 2-1……2-5* indicate the fractions from top to bottom. 2-3 was the fraction at the interface and abundant of purified OSs. All the fractions were loaded as equal proportion and detected by anti-Hsp90α and anti-Thr5/7 phosphorylated Hsp90α antibodies on western blot. **B**. Immunofluorescence staining of purified OSs. The bar is 2.5 μm. Purified OSs were stained for Thr5/7 phosphorylated Hsp90α and acetylated α-tubulin. **C**. Immunoprecipitation of Hsp90α by anti-Thr5/7 phosphorylated Hsp90α antibody from crude or purified OSs. The crude and purified OSs were dissolved in 0.5% Triton X-100 immunoprecipitation buffer (20mM Hepes PH7.4, 150mM NaCl, 0.5% TritonX-100, 1mM EDTA and 10% glycerol) and anti-Thr5/7 phosphorylated Hsp90α antibody was added to incubate at 4℃ overnight. Protein A/G magnetic beads were used to pull down the proteins. The immunoprecipitated samples were loaded and detected by anti-Thr5/7 phosphorylated Hsp90α antibody on western blot. *Total*, total OSs lysate; *Input*, proteins extracted from the OSs; *IP*, immunoprecipitated proteins by anti-pHsp90α antibody; *Sup*, supernatant after immunoprecipitation; *Pre*, the precipitated proteins of OSs that were insoluble in 0.5% Triton X-100 immunoprecipitation buffer. **D**. Coomassie blue staining of proteins immunoprecipitated by anti-Thr5/7 phosphorylated Hsp90α antibody in polyacrylamide gels. Arrows indicate the proteins identified by mass spectrometry.

Reference

Liu, Q., Tan, G., Levenkova, N., Li, T., Pugh, E.N., Rux, J.J., Speicher, D.W., and Pierce, E.A. (2007). The proteome of the mouse photoreceptor sensory cilium complex. Molecular & Cellular Proteomics *6*, 1299-1317.
